# Novel Vaccines and Drugs That Target the Surface Glycoproteins of Influenza Viruses, RSV, Parainfluenza Viruses, and SARS-CoV-2

**DOI:** 10.3390/v14061160

**Published:** 2022-05-27

**Authors:** Charles J. Russell, Elena A. Govorkova

**Affiliations:** Department of Infectious Diseases, St. Jude Children’s Research Hospital, 262 Danny Thomas Place, Memphis, TN 38105-3678, USA

## 1. Introduction

Newly emerging and seasonal respiratory viruses have a great impact on public health. Pandemic viruses like SARS-CoV-2 and emerging influenza spread rapidly across the globe and cause significant morbidity, mortality, and economic loss. Seasonal respiratory viruses such as influenza A (H1N1 and H3N2), influenza B, RSV, and the parainfluenza viruses annually cause illness, hospitalization, and mortality. This Special Issue of *Viruses* contains an original article on dose sparing of alum-adjuvanted seasonal influenza vaccines in the pediatric population and seven review articles on recently developed vaccines and drugs that target influenza virus HA and NA, RSV F and G, parainfluenza virus F and HN, and SARS-CoV-2 S glycoproteins. Novel vaccination strategies include antigen stabilization, antigen optimization, adjuvants, and eliciting immunity in high-risk populations. Novel antiviral strategies include the development of monoclonal antibodies and small-molecule drugs that target viral attachment, viral fusion-mediating, and receptor-destroying proteins. Mechanisms of drug resistance, and how to manage it, are also reviewed.

While seasonal and pandemic respiratory viruses continue to impact public health and the economy, recently developed strategies to control their diseases are promising. For example, the health, social, and economic toll of COVID-19 has been met with the remarkably fast development and implementation of novel vaccines and vaccine platforms to control SARS-CoV-2. SARS-CoV-2 is a positive-strand RNA virus from the *Coronaviridae* family. The remainder of the viruses covered in this Special Issue are negative-strand RNA viruses from the virus families *Orthomyxoviridae* (influenza A and B viruses), *Pneumoviridae* (respiratory syncytial virus, or RSV, and human metapneumovirus, or HMPV), and *Paramyxoviridae* (the human parainfluenza viruses, or HPIVs). The fusion glycoproteins of these viruses are the primary antigens of these viruses and have essential roles in viral entry. Therefore, these glycoproteins are the key target in most vaccines and have been a target for some antiviral drugs.

As reviewed in Narkhede et al. [1], these viruses contain a structural class I fusion-mediating glycoprotein that has a metastable prefusion conformation. The fusion proteins of these viruses are as follows: influenza A and B virus (hemagglutinin, HA); RSV, HMPV, and the HPIVs (fusion, F); and SARS-CoV-2 (spike, S). Upon activation, the fusion protein refolds into a hairpin structure that mediates the fusion of the viral envelope and a host cell membrane. As the prefusion conformations of these proteins in their native structure are relatively unstable, antigen engineering efforts often include mutational strategies to lock in the prefusion structure.

Small-molecule antivirals have been developed against structural features of the fusion glycoproteins, but this strategy has yielded only moderate success because of the mutability of the targeted structural motifs. Limited therapeutic success has come from the use of humanized and human monoclonal antibodies (mAbs).

The envelope glycoproteins have other functions in the virus life cycle that can be targeted by vaccines and antivirals. Before being triggered by endosomal low pH to cause membrane fusion during viral entry, the influenza virus HA protein binds sialic acid-containing receptors. Natural immunity and many vaccines against influenza viruses elicit antibody responses predominantly directed against the receptor-binding head of the HA protein. Some universal influenza vaccines in development target the more highly conserved stalk (also known as the stem).

The influenza virus neuraminidase (NA) protein enzymatically cleaves sialic acid to avoid virion aggregation and reduce superinfection. NA inhibitors are an important class of anti-influenza control. RSV and HMPV have an additional G glycoprotein with receptor-binding activity. CX3CR1 and IGF1R have been identified as RSV entry receptors and may be targeted with antivirals. The HPIVs have a hemagglutinin-neuraminidase (HN) protein that binds and cleaves sialic acid, and small-molecule therapeutics such as DAS181 have been developed that disrupt this binding. The SARS-CoV-2 S protein binds ACE2 receptors in addition to causing membrane fusion, and vaccine and drug development efforts have focused on disrupting this protein-protein interaction.

The authors highlight some of the greatest challenges in this field including few licensed vaccines and drugs currently available, reinfection, antigenic drift, and the emergence of drug-resistant variants.

## 2. Influenza Viruses

The HA and NA surface glycoproteins are the major antigenic determinants of influenza A and B viruses, and most vaccine development is focused on these proteins. Seasonal influenza vaccines currently licensed include trivalent and, beginning with the 2013–2014 season, quadrivalent inactivated influenza vaccine (IIV), live attenuated influenza vaccine (LAIV), and recombinant vaccine based on HA protein. However, influenza vaccine efficacy at preventing illness has averaged 40% over the past two decades, ranging from 10% (2004–2005) to 60% (2010–2011). Thus, the main challenge of seasonal influenza vaccines is boosting their effectiveness. Part of this challenge is selecting H1N1, H3N2, and B vaccine strains that both antigenically match circulating strains and produce high vaccine yields. An additional challenge is antigenic drift. The animal reservoir of influenza viruses is wild aquatic birds, and numerous strains to which humans are immunologically naïve circulate in domestic poultry, swine, and other animals to which humans are exposed. Therefore, increasing efforts have focused on developing universal influenza vaccines that are broadly protective against drifted seasonal strains and/or emerging strains of pandemic potential.

The review article by Tripp and Stambas [2] provides a general overview of vaccines being developed against all of these respiratory viruses, including influenza viruses. The review by Narkhede et al. [1] describes recent advances in computational structure-based design of glycoprotein-focused vaccines, including the influenza HA protein. Nagashima and Mousa [3] review efforts to expand the breadth of protection of HA-based vaccines. This includes developing HA antigens that display conserved epitopes from multiple influenza viruses and those that broaden polyclonal responses by other mechanisms including the use of adjuvants. Expanded discussions of successes using mosaic and computationally optimized broadly reactive antigens (COBRAs) are described [3]. Other strategies for developing universal influenza vaccines by focusing on responses to the stalk include headless HA vaccines, chimeric HA vaccines, and hyper-glycosylated antigens [1,3].

Increasing influenza vaccine efficacy requires eliciting protective immunity in vulnerable populations. In the review article by Wiggins et al. [4], the authors describe how high-risk populations are more susceptible to increased disease severity and decreased vaccine efficacy. These include the elderly, obese, and those with comorbidities such as type 2 diabetes mellitus. High-risk populations differ in their immunological responses to influenza vaccination. This is outlined in Table 1 of Wiggins et al. [4] and described in the following text in great detail with respect to adipose tissue composition, dendritic cells, B cells, T cells, and metabolic markers. Other intrinsic and extrinsic host factors that decrease vaccine responses to influenza vaccination include immunodeficiencies, other comorbidities, immunosenescence, undernutrition/overnutrition, and sex biases. Finally, various strategies to increase vaccine effectiveness in high-risk populations are described. For example, needle length can be optimized to minimize adverse reactions in the obese. Moreover, universal influenza vaccine antigens, adjuvants, and delivery platforms can be developed to overcome immunosenescence, reduced class switching, and immune imprinting.

The pediatric population reacts differently to influenza vaccines than adults. To enhance influenza vaccine immunogenicity in pediatric patients while maintaining safety, Vajo et al. [5] have studied dose reduction of an egg-grown whole virion, formaldehyde-inactivated, trivalent, aluminum phosphate adjuvanted vaccine (FluArt). The authors conducted a clinical trial of 120 healthy volunteers in cohorts aged 3–11 and 12–18, which received doses of 3 µg and 6 µg HA protein, respectively. Twenty-one days post-vaccination, immunogenicity was assessed using peripheral blood samples to measure seroconversion (≥4-fold increase in HI antibody titer), geometric mean titer (GMT) ratio after and before vaccination, and seroprotection as defined as the percentage of subjects with an hemagglutination inhibition (HAI) titer ≥40. A total of 80–97% of the participants in the study met the criteria of seroprotection, and no severe adverse events were reported. The study did not address the relative importance of antigen versus adjuvant and only included white Caucasians. Overall, the vaccine was shown to be immunogenic and safe in the study.

The review article by Bai et al. [6] describes antiviral drugs targeting the NA and HA glycoproteins of influenza viruses. Four NA inhibitors are in use in various countries: oseltamivir, zanamivir, peramivir, and laninamivir (approved only in Japan). NA inhibitors have been shown to be effective at decreasing disease duration and severity if first administered within 48 h of symptom onset. Resistance to NA inhibitors may occur and is monitored by the World Health Organization (WHO) Global Influenza Surveillance and Response System (GISRS) Expert Working Group for Surveillance of Antiviral Susceptibility. The review by Bai et al. describes mechanisms and key NA substitutions involved in the emergence of resistant variants and points out different patterns of resistance under specific drugs [6]. The authors also describe small molecule inhibitors targeting HA glycoprotein including existing (arbidol) and newer drugs (tert-butyl hydroquinone, flufirvitide-3, sialylglycopolymers, dendritic sialosides, and sialic acid-containing liposomes). The modes of action of HA inhibitors are to interfere with HA binding with cellular receptors or fusion, therefore, these inhibitors target the globular head, containing the receptor-binding domain, or the stalk, containing the fusion peptide, respectively. Computational design has assisted in the development of HA protein inhibitors that disrupt protein-protein interactions and, thereby, inhibit receptor binding and/or membrane fusion [1]. However, targeting the HA protein with small-molecule drugs remains challenging due to the emergence of drug-resistant substitutions, which is more likely when targeting broad structural features as opposed to an enzyme active site. Overall, clinical use of HA inhibitors is limited, partially due to the continued evolution and antigenic diversity of HA globular head and the emergence of fully fit resistant variants.

## 3. Pneumoviruses and Paramxyoviruses

While numerous vaccines against RSV, HMPV, and the HPIVs are in preclinical development, no licensed vaccines are available for these leading causes of respiratory disease in infants, young children, the elderly, and immunocompromised. Hospitalization rates for children under five infected with RSV, HMPV, HPIV1, HPIV2, and HPIV3 are approximately 77 per 1000 infected, collectively exceeding the hospitalization rate of influenza viruses. Russell and Hurwitz [7] review the development of the Sendai virus (SeV) as a vaccine vector that expresses envelope glycoproteins from pneumoviruses and paramyxoviruses in infected respiratory cells. An overview of the taxonomy, phylogeny, genome and virion structure, replication mechanisms, and envelope glycoprotein structures of the F protein, HN protein, and G protein is provided. After presenting the history of past and current RSV, HMPV, and HPIV vaccine candidates, the authors describe in greater detail the work of their research team to develop SeV as a pediatric respiratory vaccine platform. SeV is the murine counterpart of HPIV1, and the vaccine vector has been shown to be well tolerated in cotton rats, nonhuman primates, and human clinical trials. Genes are ordered in tandem in the SeV genome (N-P-M-F-HN-L), and most of the SeV vaccines have the foreign glycoprotein inserted between the F and HN genes. Modification of the gene insertion site and gene start sequence upstream of the SeV F gene has been used to modulate vector replication and foreign gene expression. Vaccines investigated preclinically in animal models express RSV F (full-length and secreted), RSV G (full-length and secreted), HMPV F (secreted), HPIV3 F, and HN (both full-length), and HPIV2 HN (full-length). In cotton rats, all of the vaccines elicit protective B- and T-cells responses without inducing pathology. The candidate that has advanced to human clinical trials, SeVRSV (rSeV that expresses full-length RSV F protein) has been shown to induce protective responses in African green monkeys and to be well tolerated in seropositive human adults. In addition to SeV, other vectors for pneumoviruses and paramyxoviruses include PIV5, measles virus, alphaviruses, and coronaviruses. Advantages of SeV are that it elicits cross-protective responses against HPIV1, can be produced at high levels in embryonated hen eggs or mammalian cell cultures, is naturally attenuated in humans, has demonstrated safety in humans, induces robust mucosal and systemic B- and T-cell responses, and elicits potent and durable immunogenicity. A limitation of SeV is that its vectored vaccines are best suited for the pediatric population because of its cross-reactivity with HPIV1.

Challenges to RSV vaccine development have included enhanced respiratory disease (ERD) after vaccination of immunologically naïve infants and children with formalin-inactivated RSV (FI-RSV) vaccine in the 1960s and the lack of an ideal animal model for RSV. The RSV F protein is the primary vaccine target as this glycoprotein is conserved between RSV strains and provides protection against challenges. Vectored RSV F vaccines may include full-length, wildtype F protein, while ectodomain protein constructs appear to benefit from stabilizing mutations that maintain critical prefusion epitopes [1]. An additional strategy for de novo design of soluble RSV F antigens is using scaffold proteins to graft key epitopes of validated neutralizing mAbs.

Tripp and Stambas [2] also provide a review of novel drugs against respiratory viruses. These include virus entry inhibitors, protease and transcriptase inhibitors, and virus particle-formation inhibitors. Palivizumab, a humanized IgG mAb that binds prefusion F protein, is clinically effective at preventing RSV disease in premature infants and children <2 with chronic lung or heart disease.

## 4. SARS-CoV-2

A wide variety of SARS-CoV-2 S protein vaccines have been developed and several have shown clinical effectiveness in widespread global Phase III clinical trials and subsequent emergency usage. The most promising approach is nucleoside-modified mRNA lipid nanoparticles vaccines. mAbs such as Regeneron (casirivimab and imdevimab) have been used to reduce the disease severity of COVID-19 [1]. Other anti-S protein inhibitors in development bind to the S protein and inhibit ACE2 binding, membrane fusion, or cleavage of polyproteins required for SARS-CoV-2 replication such as the viral proteases 3CLpro and PLpro. The major scientific challenge of targeting envelope glycoproteins with small-molecule drugs and mAbs is resistance. Therefore, some recent efforts have focused on structural motifs that are highly conserved or utilize a cocktail of neutralizing antibodies (nAbs) rather than monotherapy. Hurt and Wheatley [8] review the role of SARS-CoV-2 nAbs in the clinical management of COVID-19 and provide an overview of recent randomized controlled trial data evaluating nAbs in the ambulatory, hospitalized, and prophylaxis settings. Emerging data suggest nAbs are particularly effective in preventing patients with risk factors from progressing to severe disease requiring hospitalization. The authors also discuss the challenges associated with the use of nAbs. These include the emergence of escape mutants and efficacy against antigenically different variants, cost/access, large-scale manufacturing and storage, and mode of administration.

## 5. Conclusions

Respiratory pathogens continue to evolve, cause pandemics, and circulate seasonally. Targeting the envelope glycoproteins of these viruses has significantly reduced disease burden. Antivirals targeting the envelope glycoproteins have had limited success including the use of influenza virus NA inhibitors and SARS-CoV-2 mAbs. The key challenge is sequence drift of the envelope glycoproteins, which reduces vaccine efficacy and leads to antiviral drug resistance. Two counterstrategies to antigenic drift are developing novel technologies to rapidly update the virus strain and to develop novel antigens and adjuvants to focus vaccine responses on less-mutable epitopes.
